# Inhibition of Rho-kinase improves response to deep inspiration in ovalbumin-sensitized guinea pigs

**DOI:** 10.22038/ijbms.2020.46258.10683

**Published:** 2020-12

**Authors:** Saeed Pazhoohan, Ehsan Aref, Leila Zare, Samaneh Dehghan, Mohammad Javan, Sohrab Hajizadeh, Mohammad Reza Raoufy

**Affiliations:** 1 Department of Physiology, Faculty of Medicine, Arak University of Medical Sciences, Arak, Iran; 2 Department of Physiology, Faculty of Medical Sciences, Tarbiat Modares University, Tehran, Iran

**Keywords:** Airway smooth muscle, Asthma, Bronchodilation, Deep inspiration, Rho-kinase

## Abstract

**Objective(s):**

The modulatory effect of deep inspiration (DI) on airway constriction is impaired in asthma. However, mechanisms underlying this impairment are not clear. Since there is evidence indicating that Rho-kinase activation mediates force maintenance under oscillatory strain, we investigated the impact of Rho-kinase inhibition on the bronchodilatory effect of DI in ovalbumin (OVA) sensitized guinea pigs.

**Materials and Methods:**

forty-eight male Dunkin Hartley guinea pigs were divided into 8 groups including saline/ constant, saline/DI, OVA/constant, OVA/DI, Rho-I/OVA/constant, Rho-I/OVA/DI, OVA-Rho-I/MCh/constant, and OVA-Rho-I/MCh/DI. Animals were subjected to 12 inhalations of OVA or saline aerosol. Guinea pigs in Rho-I/OVA/constant or DI groups were treated with the Rho-kinase inhibitor (Rho-I) (Y-27632, 1 mM aerosols) prior to the last 8 allergen inhalations and OVA-Rho-I/MCh/constant or DI groups received Y-27632 at the end of allergen sensitization protocol before methacholine challenge. The bronchodilatory effect of DI in guinea pigs that were exposed to methacholine was assessed by using an animal ventilator. The bronchodilatory effect was assessed using several parameters: the airway pressure maintenance, airway pressure recovery, and decline of airway pressure.

**Results:**

Results indicated that application of Y-27632 prior to methacholine challenge reduces the airway smooth muscle ability to maintain pressure and also causes further decline in airway pressure in OVA-sensitized animals undergone DI. However, the inhibition of Rho-kinase before OVA inhalations had minimal effect.

**Conclusion:**

We propose that alteration of Rho-kinase signaling pathway may be one of the mechanisms underlying the impairment of DI-induced bronchodilation in OVA-sensitized guinea pigs.

## Introduction

Bronchoconstriction developed during airway smooth muscle (ASM) contraction in response to spasmogen agents. Airway narrowing was abolished or reduced with deep inspiration (DI) in healthy subjects (1). These effects were largely diminished in asthmatic patients (2). 

Exaggerated airway narrowing during an asthma attack is related to excessive ASM contraction (3). However, it was not clear whether an alteration in the intrinsic contractility of ASM caused the exaggerated airway narrowing or additional factors such as airway remodeling could lead to excessive contraction of the airway without an enhancement of ASM contractility (4). Another study demonstrated that the mechanical strain of ASM in asthmatic subjects induces smaller reduction in isometric force and greater force recovery in comparison to that of healthy subjects. This evidence proposes that there exists a difference between asthmatic and healthy ASMs in mechanical properties (5). However, the mechanism underlying force maintenance by ASM in a dynamic condition is still not clear.

Another study on isolated lungs showed that internal factors related to ASM could be involved in the bronchodilatory effect of DI (6). The reports declared that Rho-kinase signaling was important in tonic phasic contraction and contractility of gastrointestinal smooth muscles. This evidence proposed that the activation of Rho-kinase mediates force maintenance in smooth muscles (7, 8), and a similar role has been suggested for Rho-kinase in ASM by Lan *et al* (9). They showed that inhibition of Rho-kinase reduces the ability of ASM to maintain force under oscillatory strain in association with a decrease in the mass of myosin filament (9). Although previously it was suggested that the detachment of actin-myosin cross-links was involved to some degree in force decrease due to oscillatory strain (10), the decrease in mass of myosin filament and slow force regeneration indicated the involvement of other mechanisms such as fluidization response that was previously described (11, 12). It was suggested that fluidization response could implicate the depolymerization of myosin filaments, which in turn results in change of ASM force (9, 13). 

Generally, we assumed that ASM remodeling induced by increased Rho-kinase pathway activity in ovalbumin (OVA) sensitized animals contributes to the attenuation of the DI bronchodilatory effect. Therefore, in the present study, we investigated the effect of Rho-kinase inhibition during the asthma sensitization protocol on the bronchodilatory effect of DIs. Moreover, increase of Rho-kinase pathway activity by increasing tone and stiffness of ASM due to alteration in fluidization response could impair the bronchodilatory effect of DI in asthma. Thus, we examined the impact of Rho-kinase inhibition before methacholine challenge on the bronchodilatory effect of DI in OVA-sensitized guinea pigs.

## Materials and Methods

***Animals***

All experiments were approved by the Ethics Committee of Faculty of Medical Sciences, Tarbiat Modares University (IR.TMU.REC.1394.028). Forty-eight male Dunkin Hartley guinea pigs, weighing 350±500 g, were purchased from the Razi Institute, Karaj, Iran. Animals were housed in rooms with controlled temperature (22±1 °C), and a 12:12 hr light: dark cycle with free access to food and water.

***Antigen sensitization protocol***

Animals were placed in a plexiglass box (40 × 20 × 20 cm) and subjected to aerosol of OVA for 15 min using a compressor nebulizer (Pari, Starnberg, Germany). This protocol was repeated every 3 days for 5 weeks (totally 12 times). In order to avoid tolerance induction, the OVA was applied in incremental doses (1, 2.5, 5, and 10 mg/ml in NaCl 0.9%) (14, 15). Control animals were subjected to the same protocol using only normal saline

***Experimental groups***

**Figure 1:**
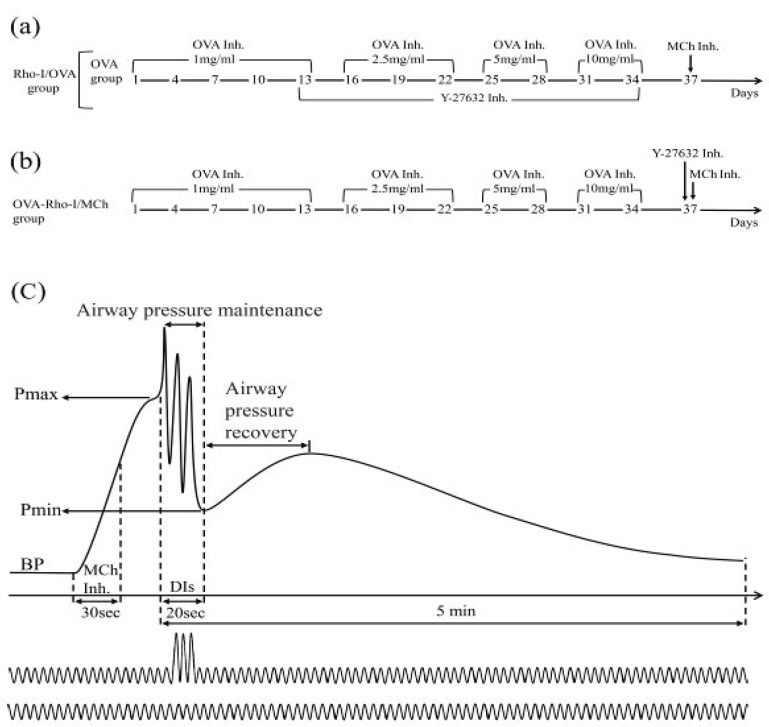
(a) Experimental protocols for describing OVA-sensitization and Rho-kinase inhibitor treatment prior to the last 8 OVA inhalations; (b) Experimental protocols for describing Rho-kinase inhibitor treatment before MCh challenge; (c) Experimental protocols for demonstrating airway pressure peak, pressure maintenance (underlie DIs), pressure recovery

Guinea pigs were randomly divided into 8 groups (n=6 in each group). Bronchodilatory effect of DI was investigated 72 hr after the last antigen or saline inhalation. Experimental groups included: OVA/constant and OVA/DI groups, animals in these groups received OVA aerosol using sensitization protocol; Rho-I/OVA/constant and Rho-I/OVA/DI groups, in these groups, animals were sensitized with OVA aerosol using sensitization protocol and they received inhalation of Rho-kinase inhibitor (Rho-I) (1 mM) (Y-27632, Apexbio, USA) 10 min before the last eight OVA exposures for 6 min (Figure 1a); OVA-Rho-I/MCh/constant and OVA-Rho-I/MCh/DI guinea pigs in these groups were sensitized by OVA then they received inhalation of Rho-I (1 mM) for 3 min, 10 min before MCh challenge (Figure 1b); saline/constant and saline/DI animals in these groups only received saline inhalation same as the protocol used for OVA administration. In constant groups, animals were just ventilated by constant rate and volume ventilation, while in DI groups animals were also subjected to DIs (Figure 1c). Also, the effect of OVA sensitization on ROCK I expression was investigated in lung tissue of guinea pigs that received only OVA or saline.

***Quantitative real-time RT-PCR***

Real-time RT-PCR was used to investigate the mRNA expression of ROCK I in the lung tissue. GAPDH RNA was chosen as an internal control. Approximately 100 mg of the right lung was collected and immediately frozen in liquid nitrogen and then stored at −80 °C until RNA extraction. The frozen lung tissues were finely ground in liquid nitrogen using a mortar and pestle. Total RNA was extracted using TRIzol reagent (Invitrogen). The First-strand of cDNA was synthesized using a cDNA reverse transcription kit (Aryatous Biotech, Tehran, Iran), and quantitative real-time PCR was performed using QuantiFast SYBR Green PCR Kit. The PCR cycle conditions were as follows: incubation at 95 °C for 5 min, followed by 40 cycles of denaturation step at 95 °C for 30 sec, annealing step at 60 °C for 30 sec, and extension step at 72 °C for 30 sec. Oligonucleotide primers used for PCR were as follows:

Guinea pig GAPDH, F: CCAGGGCTGCTTTCATGTCT, R: GATCTCGCTCCTGGAAGATGG. NM_001172951.1.

Guinea pig ROCK I, F: TGCTACTGGATAAATCTGGA, R: ATAACCATCACCACCTTGAG

XM_003474112.3.

***Animal preparation and ventilation***

72 hr after the last aerosol exposure, animals were anesthetized by urethane (1.5 g/kg), tracheostomized, and the ventilation was through an animal ventilator (Harvard Apparatus, Holliston, MA) at a tidal volume of 1.0 ml/100 g body weight and frequency of 60 breaths/min.

***Airway pressure measurement***

Figure 1c illustrates the protocol operation. Following 5 min ventilation and stabilization, basal airway pressure (BP) was measured. Then a single dose of MCh was applied (Provocholine, Canada) (1.6 mg/ml) by compressor nebulizer for 30 sec. In DI subgroups, when airway pressure reached maximum pressure (pmax), animals were subjected to three DIs (interval between each DI was 6 sec, volume was 2.0 ml/100 g body weight and duration of each DI was 0.7 sec) totally during 20 sec. For quantify of peak airway pressure, the ventilator’s airway pressure output was recorded at a sampling rate of 1 kHz (Powerlab, AD Instruments, Australia). Airway pressure maintenance under DI and constant conditions were computed by recording alteration in airway pressure within 20 sec after the Pmax. After the last DI, the minimum recorded pressure was considered as the Pmin. Airway pressure recovery after cessation of DIs was monitored for an additional 60 sec. To quantify the recovery of airway pressure, we divided each airway pressure measured at intervals of 10 sec by the Pmin. Airway pressure decline in constant ventilation and DI conditions were computed by recording alteration in airway pressure during 5 min after the Pmax. In both DI and constant conditions, airway pressure was calculated at one-minute intervals. The guinea pigs were sacrificed by exsanguination under anesthesia. The data of the OVA/saline group were not shown as no significant differences were observed between this group and the OVA group (15).

***Statistical analysis***

The GraphPad Prism was used for statistical analysis. Data were presented as Mean±SEM. The differences in airway pressure among groups were assessed using one-way ANOVA followed by Bonferroni’s test. Analysis of maintenance, recovery and attenuation of airway pressure were performed using repeated-measures two-way ANOVA followed by Bonferroni’s test. Analysis of mRNA expression was performed using unpaired t-tests. The significance level was set at 0.05.

## Results

***Expression of ROCK I mRNA in lung tissue***

**Figure 2:**
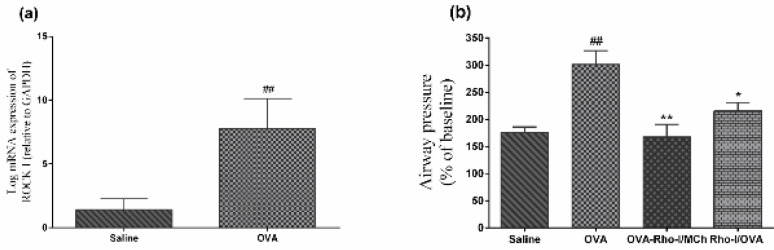
(a) mRNA expression of ROCK I in the lung of OVA and saline groups. (b) Effect of Rho-kinase inhibition on airway responsiveness in response to MCh. ## *P*<0.01 compared with saline; * *P*<0.05 and ** *P*<0.01 compared with OVA; analyzed by unpaired t-tests and one-way ANOVA, with the Bonferroni post-test. Data are presented as mean±SEM (n=6). OVA: Ovalbumin; Rho-I: Rho-kinase inhibitor

Repeated exposure to OVA significantly increased mRNA expression of ROCK I (*P*<0.01) in the lung tissue (Figure 2a).

***Effect of Rho-kinase inhibition on airway responsiveness in response to MCh***

Airway responsiveness to MCh challenge was determined by evaluating changes in airway pressure and quantified as the percentage of maximum airway pressure (Pmax) evoked by MCh to basal pressure (BP); (Pmax/BP)×100. Airway responsiveness to MCh in the OVA group was significantly higher than in the saline group (*P*<0.01). Inhibition of Rho-kinase in OVA-Rho-I/MCh (*P*<0.01) and Rho-I/OVA (*P*<0.05) groups resulted in reduction of airway responsiveness in response to MCh level compared with the OVA group (Figure 2b).

***Effect of Rho-kinase inhibition on airway pressure maintenance during constant and DI conditions***

**Figure 3:**
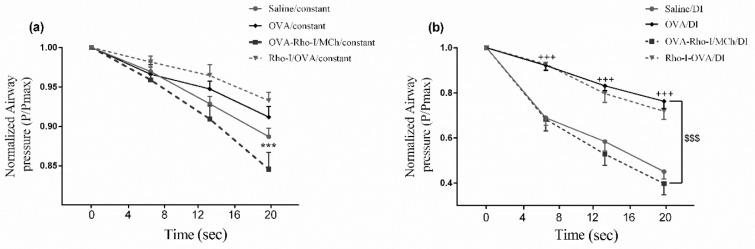
Effect of Rho-kinase inhibition on airway pressure maintenance. (a) during constant condition, (b) during DI conditions. ***P<0.001 compared with OVA/constant; +++ *P*<0.001 compared with saline/DI; $$$ *P*<0.001 compared with OVA/DI; analyzed by repeated measure two-way ANOVA, with the Bonferroni post-test. Data are expressed as mean±SEM (n=6). OVA: Ovalbumin; Rho-I: Rho-kinase inhibitor, DI: Deep inspiration

In order to quantify the pressure maintenance during period of 20 sec, airway pressure values were plotted at intervals of 6 sec, as shown in Figure 3a. Airway pressure maintenance was computed by dividing airway pressure obtained in each interval by Pmax. There was no significant difference in airway pressure maintenance between saline/constant and OVA/constant. Inhibition of Rho-kinase just before MCh challenge (OVA-Rho-I/MCh/constant) significantly depressed pressure maintenance compared with OVA/constant (*P*<0.001). However, inhibition of Rho-kinase during sensitization protocol (Rho-I/OVA/constant group) had no significant effect on pressure maintenance (Figure 3a).

To assess the ability of ASM to maintain airway pressure against mechanical strain, DI was applied by 6 sec intervals, during a period of 20 sec. Airway pressure maintenance was measured following each DI by the Pmax. There was a significant difference between saline/DI and OVA/DI groups (*P*<0.001). Inhibition of Rho-kinase in the OVA-Rho-I/MCh/DI group significantly reduced pressure maintenance compared with the OVA/DI group (*P*<0.001). However, there was no significant difference in airway pressure maintenance between Rho-I/OVA /DI and OVA/DI groups (*P*>0.05) (Figure 3b).

***Effect of Rho-kinase inhibition on airway pressure recovery after DI***

**Figure 4:**
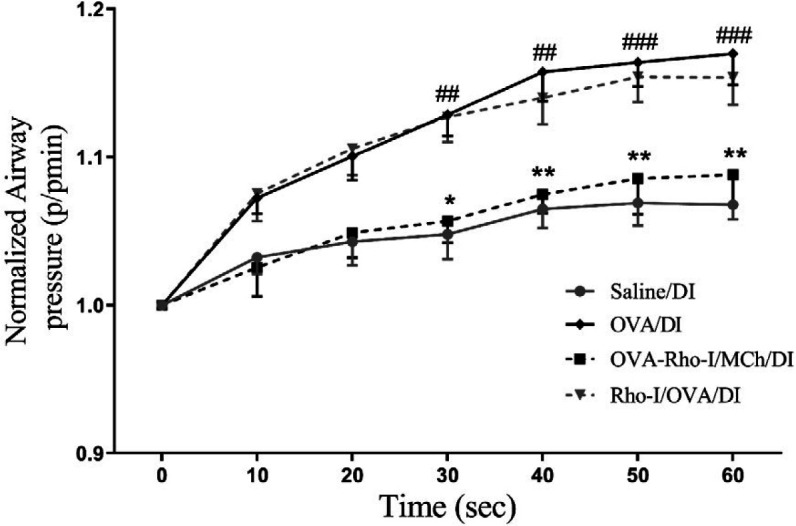
Effect of Rho-kinase inhibition on airway pressure recovery after DI. ## *P*<0.01 and ### *P*<0.001 compared with saline/DI; * *P*<0.05 and ** *P*<0.01 compared with OVA/DI; analyzed by repeated measure two-way ANOVA, with the Bonferroni post-test. Data are presented as mean±SEM (n=6). OVA: Ovalbumin; Rho-I: Rho-kinase inhibitor and DI: Deep inspirations

Regeneration of airway pressure following DIs is plotted in Figure 4. To quantify the recovery of airway pressure, we divided each airway pressure measured at intervals of 10 sec from Pmin, for a period of 60 sec, by the Pmin. OVA/DI group displayed significantly higher levels of airway pressure redevelopment compared with the saline/DI group (*P*<0.01). OVA-Rho-I/MCh/DI group exhibited a reduction in the ability of ASM to redevelop pressure following DIs compared with OVA/DI (*P*<0.05). However, no significant difference was found in the recovery of airway pressure between Rho-I/OVA /DI and OVA/DI groups. 

***Effect of Rho-kinase inhibition on the decline of airway pressure under constant and DI conditions***

**Figure 5:**
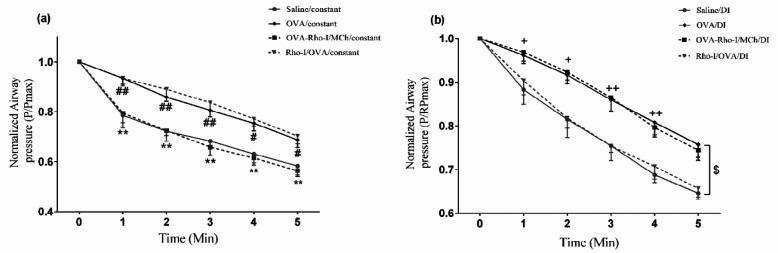
Effect of Rho-kinase inhibition on the reduction of airway pressure. (a) under constant conditions, (b) during DI conditions. # *P*<0.05 and ##* P*<0.01 compared with saline/constant; ** *P*<0.01 compared with OVA/constant; + *P*<0.05 and ++ *P*<0.01 compared with saline/DI; $ *P*<0.05 compared with OVA/DI; analyzed by repeated measure two-way ANOVA, with the Bonferroni post-test. Data are presented as mean±SEM (n=6). OVA: Ovalbumin; Rho-I: Rho-kinase inhibitor

To gain insight into the effect of Rho-kinase on reversibility of airway narrowing in asthma, we monitored airway pressure for 5 min from maximum responsiveness to MCh (Figure 1c). Airway pressure values are shown at 1 min intervals (Figure 5a). Airway pressure level decreased at a slower rate in OVA/constant compared with saline/constant (*P*<0.05). Inhibition of Rho-kinase in OVA-Rho-I/MCh /constant group significantly increased the rate of airway pressure attenuation compared with the OVA/constant group (*P*<0.01). However, no difference in airway pressure attenuation was found in Rho-I/OVA /constant and OVA/constant groups.

To examine the effects of DIs on the reversibility of airway narrowing to initial diameter, airway pressure values were monitored for 5 min from the peak of airway responsiveness to MCh and were plotted at 1 min intervals. Airway pressure was reduced more slowly in OVA/DI compared with saline/DI (*P*<0.05). Airway pressure reduction was significantly intensified in the OVA-Rho-I/MCh /DI group compared with the OVA/DI group (*P*<0.05). Nevertheless, no significant difference was found between Rho-I/OVA /DI and OVA/DI groups in force recovery after DIs (Figure 5b).

OVA: Ovalbumin; inh.: inhalation; AHR: Airway hyperresponsiveness; BP: basal pressure; Pmax: maximum pressure; Pmin: minimum pressure; MCh: Methacholine; DI: Deep inspirations

## Discussion

In this study, our main hypothesis was that the inhibition of Rho-kinase in OVA-sensitized guinea pigs would increase the bronchodilatory effect of DI following airway hyper-responsiveness (AHR). Consistently, the most significant finding of our results was that inhibition of Rho-kinase reverses bronchoconstriction in OVA-sensitized animals. 

Asthma is a disease characterized by specific clinical and pathological hallmarks including reversible airway narrowing, airway remodeling, inflammation, and deficiency of DI effects (2, 16, 17). Researchers have demonstrated that airway narrowing induced by MCh inhalation is diminished in non-asthmatic patients after DI (2). They revealed that lung inflation (through deep breath) had a beneficial effect on airway diameter and this effect was markedly reduced in asthma. Some studies have suggested that the impairment of DI effects could lead to AHR to a variety of stimuli in asthma (2, 16). We found that OVA sensitization in guinea pigs is associated with increase in AHR, and inhibition of Rho-kinase reduces the AHR. These findings confirmed the beneficial effects of Rho-kinase inhibition in asthmatic animal models (15, 18).

It has been documented that impairment of the bronchodilatory effect of DI could originate from the alteration of intrinsic properties of ASM in asthma (6). Besides, Kuo *et al*. have shown that ASM is submitted to length oscillation thick filaments which are labile and this lability would greatly facilitate plastic changes of ASM (13). Moreover, applying transient oscillation to smooth muscle resulted in rapid disassembly of actin cytoskeleton in fluidization response and reduced the rate of actin reassembly in solidification response (12). However, the mechanism/s underlying these responses is still poorly understood. A study recently reported that dynamic rearrangement of the contractile apparatus is a likely mechanism underlying many observed phenomena in the behavior of smooth muscle under mechanical strain (19). However, factors underlying dynamic reorganization of contractile filaments were not clear. Recently Lan *et al.* demonstrated that Rho-kinase inhibition in ASM increased contractile filament stability under length perturbation(9).

It should be pointed out that Rho-kinase inhibition after sensitization protocol could improve the bronchodilatory effect of DIs in OVA-sensitized animals. Also, Rho-kinase inhibition attenuated airway pressure recovery and maintenance following DIs. These findings were consistent with aforesaid studies (9, 20), which reported that force maintenance is regulated by Rho-kinase. A large body of evidence derived from experimental studies has demonstrated that when Rho-kinase activity is pharmacologically blocked (15, 21, 22) or genetically knocked out (23) AHR and ASM stiffness reduces. Our results revealed that expression of Rho-kinase has been increased in the lung tissue of OVA-sensitized animals. This finding was consistent with studies, that have shown up-regulation of Rho-kinase expression in ASM of asthmatic patients (24) and OVA-sensitized rats (25). In addition, we found that pretreatment with Rho-I before each OVA inhalation did not improve DIs effects. This suggests that likely overexpressed Rho-kinase signaling pathway by increasing ASM tone and stiffness was involved in exaggerated bronchoconstriction and DI defect, and Rho-inhibition before MCh inhalation with disruption in Rho-kinase signal improved the DI effect in OVA-sensitized animals.

Therapeutic relevance: undoubtedly asthma medications such as beta 2 agonists which save numerous lives each year, are not perfect. Several clinical studies investigating various measures of clinical efficacy have reported that a high percentage of asthmatic patients had suboptimal control (26). Several studies have linked beta-agonist use with adverse patient outcomes such as deterioration of asthma control (27, 28), serious asthma exacerbations (29), and loss of bronchodilation (30). However, it has been postulated that the inhibition of Rho-kinase reduced vascular smooth muscle tone, and was used for some clinical conditions such as pulmonary hypertension (31) and cerebral vasospasm (32, 33). Furthermore, Rho-kinase was involved in ASM remodeling (15), AHR (15, 22), and increasing the stability of contractile filament (9). Altogether, the results indicated that Rho-kinase could be responsible for at least a portion of pathophysiological aspect of asthma that was previously unknown; therefore, it would suggest that Rho-kinase inhibitors could be an add-on therapeutic that targets an untapped pathological change in asthma. However, more studies are still required to clarify the therapeutic effects of Rho-kinase inhibitors in asthma treatments. Also, future studies can elucidate which enzyme within the Rho-kinase signaling pathway plays a pivotal role in asthma pathophysiology.

## Conclusion

According to the results, OVA inhalation induced ROCK I expression in guinea pigs’ lung tissues and this was associated with AHR and impairment of DI bronchodilatory effect. Inhibition of Rho-kinase, both during and at the end of OVA-sensitization protocol, improved AHR, but just inhibition of Rho-kinase after sensitization (before MCh challenge) improved airway response to DI. It seems that change in Rho-kinase function in asthma might be involved in the alteration of ASM response to DI.
